# Using a Patient-Reported Outcome Measure to Assess Physical, Psychosocial, and Existential Issues in COPD

**DOI:** 10.3390/jcm13206200

**Published:** 2024-10-18

**Authors:** Henriette Darum Sørensen, Cecilie Lindström Egholm, Anders Løkke, Edina Nikolett Barna, Mie Sand Hougaard, Mette Raunkiær, Ingeborg Farver-Vestergaard

**Affiliations:** 1 REHPA, the Danish Knowledge Centre for Rehabilitation and Palliative Care, Odense University Hospital, 5800 Nyborg, Denmarkcecilie.lindstrom.egholm@rsyd.dk (C.L.E.); mette.raunkiaer@rsyd.dk (M.R.); 2 Department of Clinical Medicine, University of Southern Denmark, 5230 Odense, Denmark; 3 Department of Medicine, Lillebaelt Hospital, 6000 Vejle, Denmark; anders.lokke@rsyd.dk (A.L.); edina.nikolett.barna@rsyd.dk (E.N.B.); 4 Department of Regional Health Research, University of Southern Denmark, 5230 Odense, Denmark; 5 Department of Oncology, Gødstrup Hospital, 7400 Herning, Denmark; miehouga@rm.dk

**Keywords:** patient-reported outcomes, chronic obstructive pulmonary disease, general palliative care, needs assessment

## Abstract

**Background**: Chronic obstructive pulmonary disease (COPD) is marked by severe physical symptoms, impaired quality of life, and high psychological distress. Despite its impact, the identification of not only physical but also psychosocial and existential issues in the clinic lags behind that of other patient groups. **Methods**: This study aimed to assess physical, psychosocial, and existential issues among patients with COPD using a patient-reported outcome measure for general palliative care (the ‘PRO-Pall’) in a Danish outpatient clinic. We included 115 adults with COPD who completed the PRO-Pall either electronically or in the clinic. Sociodemographic and illness-related data were retrieved from their electronic health records. **Results**: We found that shortness of breath, tiredness, and difficulty walking were predominant physical issues. Worry about change in social roles was the most frequently reported psychosocial issue, while existential issues were reported by approximately one in ten patients. Most patients (44.5%) felt able to share their feelings with family or friends, and a majority (62.2%) felt their illness-related issues were addressed satisfactorily. Females expressed a greater need for rest and males more frequently reported intimacy issues. Higher COPD-impact on life measured by the COPD Assessment Test was associated with lower ratings on the quality-of-life item of the PRO-Pall independent of age, gender, lung function, and smoking status. **Conclusions**: Patients reported issues in physical, psychosocial, and existential dimensions of the PRO-Pall. The PRO-Pall shows potential as a broader alternative to measures that focus mainly on physical issues.

## 1. Introduction

Chronic obstructive pulmonary disease (COPD) is a leading cause of death and disability worldwide [1]. Advanced COPD is characterised by a heavy symptom burden, poor quality of life, impaired functional status, anxiety, and depression [2,3]. The unpredictable trajectory of COPD makes identification of not only physical but also psychosocial and existential issues relevant with the purpose of timely initiation of general palliative care [4,5,6].

The World Health Organization defines palliative care as an approach that prevents and relieves suffering and enhances quality of life through identification, assessment, and treatment of patients’ physical, psychological, social, and existential issues [7]. Palliative care can be delivered by specialised care units from hospitals and/or hospices, typically towards the end of life, or by non-specialised healthcare professionals in general healthcare settings. Research shows that early initiation of general palliative care for patients with COPD is beneficial [8,9,10]. However, access to palliative care for patients with COPD remains limited [11,12,13]. A Danish study by Bove et al. [2] found that specialized palliative care was only offered to few patients with COPD, with over 95% of the resources allocated to patients with cancer. However, compared to patients with lung cancer, those with COPD report more unmet needs in treatment and care, lower quality of life, and higher levels of psychological distress [14].

Barriers to timely palliative care for patients with COPD include misunderstandings about the definition and relevance of palliative care, patients’ limited awareness of the severity of their disease, and the unpredictable disease trajectory [3,4,11]. Consequently, it is recommended that palliative care for COPD should be based on needs and symptoms rather than prognosis [5].

Various approaches exist to identify patients’ care needs. In COPD, the assessment of patients’ self-reported needs are often focused solely on physical issues, e.g., the COPD Assessment Test (CAT) [15], and/or the Medical Research Council (MRC) dyspnea score [16]. Holistic assessment tools can support the identification of not only physical but also psychosocial and existential issues [3,5,12]. Individualized assessment is crucial for identifying patients’ needs, and patient-reported outcome measures (PROMs) offer a method to directly capture patients’ issues, instead of relying solely on the healthcare professionals’ assessment, which can result in under-detection [17]. In Denmark, a standardized PROM for assessing physical, psychosocial, and existential issues for general palliative care in patients with life-threatening diseases (‘PRO-Pall’) has been launched by national authorities, but it has not previously been used among patients with COPD in a real-world hospital setting.

The aim of the present study was to assess physical, psychosocial, and existential issues in a population of patients with COPD by using a PROM for general palliative care (‘PRO-Pall’).

## 2. Materials and Methods

The present study was carried out in the outpatient respiratory clinic at Lillebaelt Hospital, a mid-size hospital in the region of southern Denmark, in the period from January 2023 to January 2024.

### 2.1. Participants

Patients aged 18 or older with COPD who were able to complete the PRO-Pall questionnaire in Danish and had no significant cognitive impairments were invited to complete the PRO-Pall approximately one month before their scheduled consultation in the outpatient respiratory clinic. The PRO-Pall was distributed through the hospital’s electronic patient platform. Along with their hospital appointment invitation, patients received instructions to complete the PRO-Pall via their electronic mailbox [‘e-Boks’] and were informed that their responses would be used during the consultation. Patients who did not complete the PRO-Pall at home were invited to complete it on a tablet or paper version in the hospital waiting room prior to the consultation. During the consultation, patients were informed about the research project and provided written consent to use their PRO-Pall data in this study.

### 2.2. Data Collection

The PRO-Pall assesses physical, psychosocial, and existential issues through 24 items: 15 from the European Organization for Research and Treatment of Cancer Quality of Life Questionnaire Core 15 Palliative Care (EORTC-QLQ-C15-PAL) [18], 3 from the EORTC item library (oedema, loneliness, intimacy/sexual health) [19], 5 newly developed items (sore and dry mouth, social support, practical support, existential problems) [20], and 1 Write In Symptoms/Problems (WISP) [21] item allowing adding up to three additional, free-text issues. The complete PRO-Pall tool can be found in Table A1. In a previous feasibility study of the PRO-Pall, 79% of patients reported spending 15 min or less completing the questionnaire [20].

The COPD Assessment Test (CAT) assessed COPD-impact on life [15], and the Medical Research Council (MRC) dyspnea score [16] measured the impact of breathlessness on function. Data on sociodemographic variables (age, gender), disease-related factors (lung function, comorbidities, smoking status), and treatments (medication, oxygen, rehabilitation) were collected.

All data from individual participants in the present study were retrieved from their electronic health records.

### 2.3. Data Analysis

Analyses were performed using Stata Statistical Software 18 [22]. Skewness and kurtosis tests of normal distribution were applied along with graphical inspection of histograms and Q-Q plots. Frequencies of reported PRO-Pall items were calculated and presented as stacked bar charts. Gender differences in sociodemographic and disease-related variables were analysed with *t*-tests and *chi*^2^-tests. Gender differences in individual PRO-Pall items were analysed using Mann–Whitney *U* tests. Multiple linear regression analysis was performed with the quality-of-life item in the PRO-Pall tool [23] as the dependent variable and age, sex (male vs. female), smoking status (smoking vs. non-smoking), lung function (FEV1% pred.), and COPD-impact on life (CAT) as independent variables. The quality-of-life item was considered a continuous outcome as it was rated on a 7-point Likert scale [24,25]. *p*-values of <0.05 were considered statistically significant.

### 2.4. Ethics

The study was registered with the Records of Processing Activities in the Region of Southern Denmark regarding Research and Quality Projects (j.no. 22-4403). The Regional Committee on Health Research Ethics for Southern Denmark was informed about the study and determined that the study required no formal ethical approval (j.no. 2022000-06). The study complies with the Declaration of Helsinki, and all patients gave informed written consent to participate.

## 3. Results

A total of 123 patients accepted to participate. Data from the medical records could not be obtained for eight patients, and the remaining 115 participants were therefore included in the present study (Table 1).

### 3.1. PRO-Pall Responses

**Figure 1:**
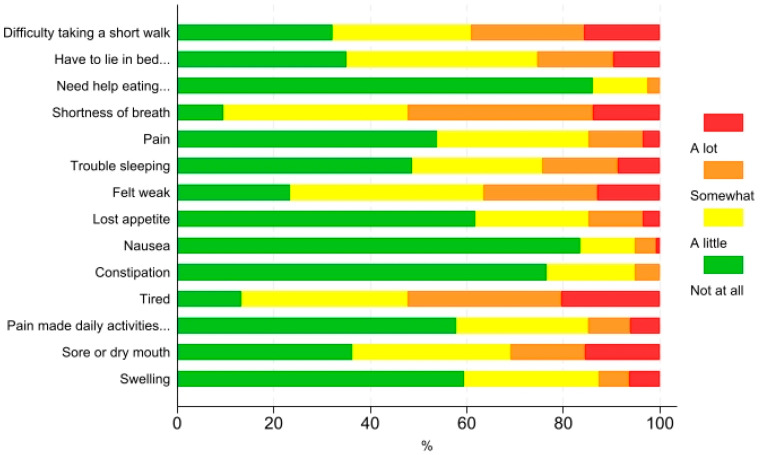
Physical issues among outpatients with COPD. Complete item descriptions are provided in Appendix A.

**Figure 2:**
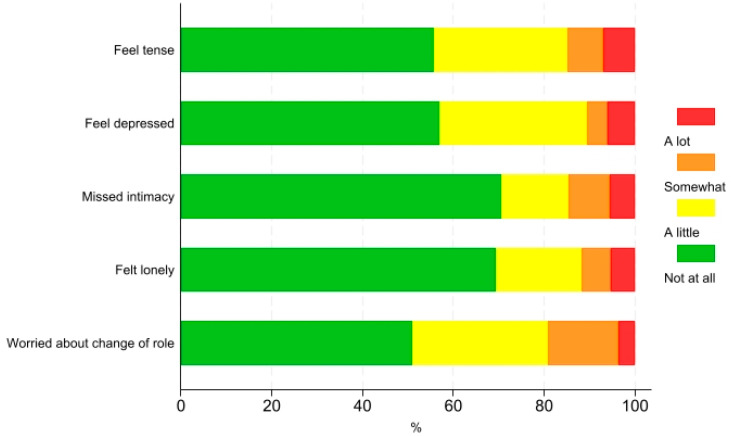
Psychosocial issues among patients with COPD.

**Figure 3:**
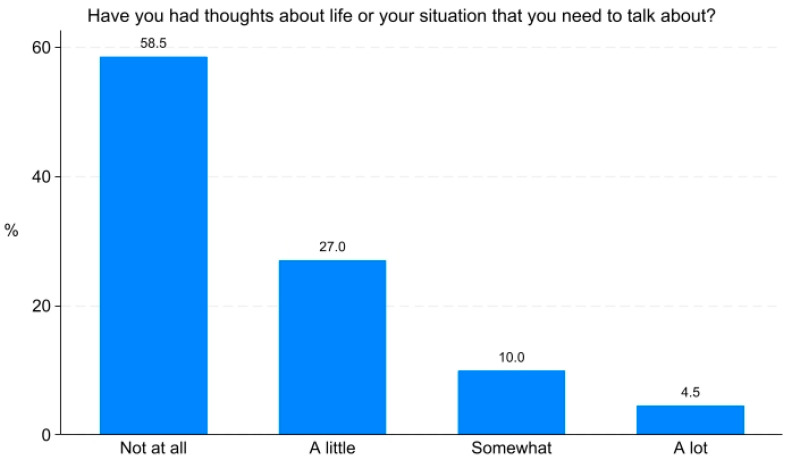
Existential issues among patients with COPD.

The physical issues that were most frequently reported as being present ‘somewhat’ or ‘a lot’ were shortness of breath (52.2%), tiredness (52.2%), and difficulty going for a short walk (39.1%) (Figure 1). The most frequently reported psychosocial issue was ‘worried about change of role’ (19.1%) (Figure 2). Existential issues were reported ‘somewhat’ or ‘a lot’ by 14.4% of the patients (Figure 3).

The PRO-Pall item ‘Have you been able to share your feelings with your family or friends as much as you would like?’, resulted in 44.5% patients responding ‘all the time’, 31.8% ‘most of the time’, 10% ‘sometimes’, 10.9% ‘rarely’, and 2.7% ‘not at all’.

When asked ‘Have the problems you have had in connection with your illness (such as financial, practical or personal) been taken care of?’, 62.2% responded that ‘problems have been taken care of/no problems’, 17.1% ‘mostly taken care of’, 9% ‘partly taken care of’, 5.4% ‘by and large not been taken care of’, and 6.3% ‘not at all taken care of’.

### 3.2. Gender Differences

**Figure 4:**
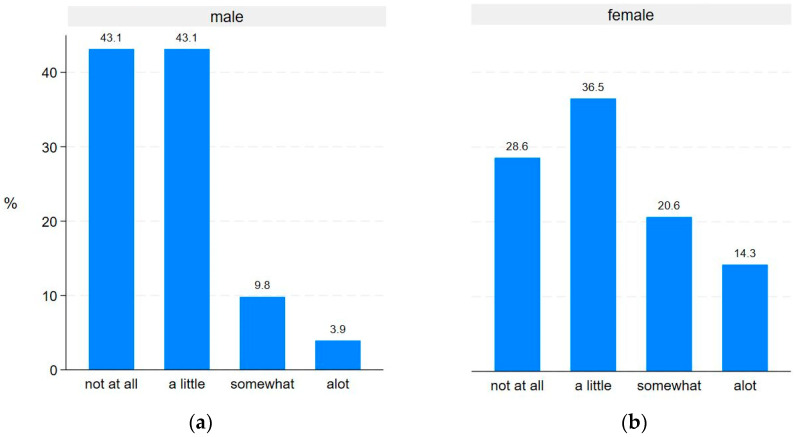
Gender differences in ‘Do you have to lie in bed or sit in a chair during the day’: (**a**) responses from male participants (**b**) responses from female participants.

**Figure 5:**
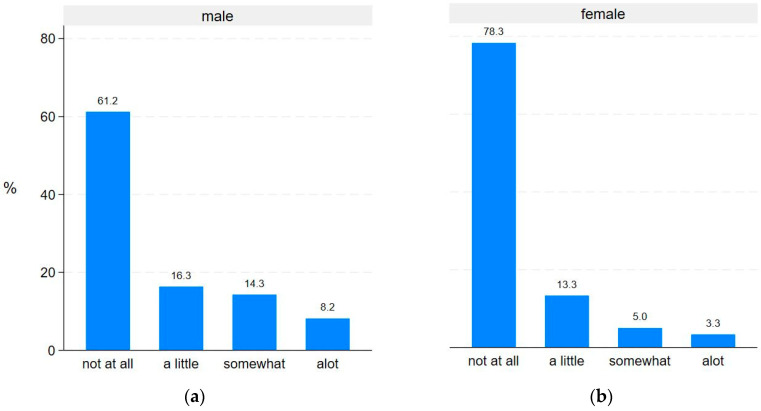
Gender differences in ‘During the past week, have you missed intimacy’: (**a**) responses from male participants; (**b**) responses from female participants.

Statistically significant differences between males and females were observed for the PRO-Pall items ‘have to lie in bed or sit in a chair during the day’ (*p* = 0.015) (Figure 4) and ‘missing intimacy’ (*p* = 0.037) (Figure 5). No statistically significant gender differences were found for the remaining PRO-Pall items.

### 3.3. Predictors of Quality of Life

The results of the multiple linear regression (Table 2) showed that the model was significant (F(5,102) = 5.78, *p* < 0.001) and explained 22% of the variance in quality of life, with an adjusted R^2^ of 0.18. COPD-impact on life measured by the CAT was a significant predictor, t(102) = −4.91, *p* < 0.001, with higher levels of COPD-impact on life being associated with lower levels of the quality-of-life item of the PRO-Pall. Age, gender, smoking status, and lung function were non-significant.

### 3.4. Additional Problems

Seventeen participants (14.8%) used the WISP item’s free-text fields and reported additional issues.

## 4. Discussion

The present study assessed physical, psychosocial, and existential issues among patients with COPD, using a PROM for general palliative care (PRO-Pall) in an outpatient hospital setting.

The most frequently reported physical issues were shortness of breath, tiredness, and difficulty with short walks. These findings align with other studies where breathlessness and its impact on functional capacity are described as the paramount symptoms by patients with advanced COPD [26]. Tiredness, as an aspect of fatigue, is commonly reported across chronic diseases among older individuals and contributes to functional limitations and impaired quality of life, especially in advanced disease stages [27].

The most frequently reported psychosocial issue in the present study was worry about changes in familial and social roles. The functional limitations of COPD can alter traditional family roles, turning partners into ‘patients’ and ‘caregivers’ [28]. Unlike cancer or heart disease, the slower progression of COPD may make these roles change less noticeably, possibly leading to passive caregiver roles. This contrasts with cancer, where partners often take active roles in medical decisions and emotional support [29]. Moreover, patients with COPD often experience a gradual shift in their social roles outside the family, influenced by societal perceptions of the illness and stigma associated with smoking, which is commonly linked to the disease [30]. This stigma can perpetuate social isolation, feelings of guilt and reluctance to seek help, further exacerbating the impact of the disease [31].

Approximately one in ten patients in the present study reported existential issues. Existential distress has been described by Vehling and Kissane [32] as a “distinct painful psychological state that results from a stressor that challenges fundamental expectations about security, interrelatedness with others, justness, controllability, certainty, and hope for a long and fruitful life”. Compared to the stressor of a cancer diagnosis, COPD could be a more diffuse and prolonged stressor, leading to gradual desensitization to existential problems. Alternatively, the low levels of existential issues in the present study could be due to the PRO-Pall item wording, i.e., ‘Have you had thoughts about life or your situation that you need to talk about?’, which may not fully capture the concept of existential distress.

In the present study, most patients felt they could share their feelings with friends and family, contrasting with studies highlighting COPD-related stigma and communication difficulties [33,34,35]. Additionally, most patients reported that their financial, practical, and personal problems were addressed, despite persistently high physical symptom levels. This may stem from optimal levels of care, or alternatively from low expectations to what can be done for COPD, or self-blame for their illness, leading to lower expectations for help. In COPD, patients may be satisfied with treatment effort, even without significant effects, as it shows something is being done to help.

We found significant gender differences in two PRO-Pall items. First, females reported a higher need for rest, potentially due to a higher prevalence of cardiovascular disease, asthma, and sleep apnea, all of which can contribute to lower energy and need for rest. However, no significant difference between males and females was found for tiredness ratings. In a cross-sectional study, Kim-Dorner et al. found that 9 out of 10 patients with COPD had at least one comorbidity and 51.7% had more than three comorbidities. They found no gender differences in the numbers of comorbidities, however they did find that males had a higher prevalence of cardiovascular comorbidities and females had a higher prevalence of asthma [36]. This difference in the two studies could possibly be explained by sampling differences across studies, with Kim-Dorner et al. also including data from primary care. Second, males reported missing intimacy more often than females. As the present study did not include a non-COPD control group, it is not clear whether this difference reflects a general difference between males’ and females’ need for intimacy, or whether it is due to COPD affecting males and females differently. According to existing research, three out of four males with COPD experience erectile dysfunction [37], which could be a factor impacting males’ engagement in intimate behaviour. Changes in relationship roles and COPD symptoms like coughing and breathlessness may also affect males and females’ intimacy needs differently [37,38]. Additionally, females more often have more external relational support [38], which may compensate for the need for intimacy with their partner.

We found that higher levels of COPD-impact on life, assessed by the COPD Assessment Test, were associated with lower rating on the quality-of-life item of the PRO-Pall, independent of age, gender, smoking status, and lung function. This suggests that the PRO-Pall could potentially be a substitute for the CAT, which mainly focus on physical issues of disease impact, with the purpose of identifying not only physical but also psychosocial and existential issues among patients with COPD.

PROMs can facilitate discussions and support the identification of patients’ issues and needs for timely palliative care initiation [39]. They can also help visualize patients’ perspectives, ensuring that treatment aligns with their needs. Given discrepancies between clinicians’ and patients’ perceptions of issues and needs [17], PROMs are crucial. However, we found that 14.8% of participants reported problems not included in the close-ended PRO-Pall items, acknowledging that no instrument can fully capture all issues that may require palliative care among patients with COPD (or any other disease) [40]. This finding underscores that PROMs should support patient–provider conversations and assessment of palliative care needs; they should not stand alone.

Healthcare providers may need additional training in order to assess whether the identified issues can be adequately treated within the clinic or if referral to specialized care units are needed. Regular PROM assessments over time and throughout disease trajectories are recommended for coherent care, though this was beyond the scope of the present study.

### Limitations

This study was conducted in a real-world hospital setting, enhancing its external validity. We used a PROM that includes assessment of not only physical but also psychosocial and existential issues. However, a number of limitations should also be noted. First, extra efforts in giving the patients opportunities to complete the PRO-Pall were provided in the present study, which might not be feasible in all clinical settings, potentially excluding those with lower literacy or emotional or technical barriers [41,42]. Second, the study’s setting, focusing on relatively ill patients, may not represent those managed in general practice or in municipalities. Moreover, the absence of a control group prevents us from drawing conclusions regarding the COPD-specificity of the findings. Third, the PRO-Pall was administered to patients one month prior to their visit in the outpatient clinic, and the reported symptoms and problems could therefore have changed in the meantime. Additionally, we did not include follow-up data, and we can therefore not make any conclusions in terms of the repeatability or long-term consistency of the procedure. Fourth, parts of the PRO-Pall tool are currently only available in Danish. Fifth, the limited sample size and multiple statistical tests may have increased the risk of Type I error.

## 5. Conclusions

Patients reported issues across physical, psychosocial, and existential dimensions of the PRO-Pall. The most common issues included shortness of breath, tiredness, difficulty with physical activities, and worry about social role changes. The PRO-Pall has potential as a broader alternative to patient-reported measures that focus primarily on physical symptoms.

## Figures and Tables

**Table 1:** Participant characteristics.

	Total	Male	Female	*p*
N	115	51	64	
Age, mean (SD)	70.7 (8.7)	70.8 (8.5)	70.6 (8.9)	0.90
FEV1% pred., mean (SD)	42.5 (14.7)	43.1 (16.0)	42.0 (13.7)	0.70
FEV1 L, mean (SD)	1.1 (0.5)	1.3 (0.5)	0.9 (0.4)	<0.001
FVC% pred., mean (SD)	67.2 (16.5)	65.9 (18.0)	68.2 (15.2)	0.49
FVC L, mean (SD)	2.3 (0.8)	2.7 (0.8)	1.9 (0.5)	<0.001
Prednisolone (synthetic glucocorticoid) within 1 month	63 (56.8%)	23 (48.9%)	40 (62.5%)	0.18
LTOT	16 (14.0%)	5 (9.8%)	11 (17.5%)	0.24
NIV at home	9 (7.8%)	4 (7.8%)	5 (7.8%)	1.00
Current smoker	40 (34.8%)	21 (41.2%)	19 (29.7%)	0.24
MRC, mean (SD)	3.2 (0.9)	3.0 (0.9)	3.4 (0.9)	0.04
CAT, mean (SD)	16.3 (6.7)	16.0 (6.5)	16.4 (6.9)	0.75
Cancer	32 (27.8%)	16 (31.4%)	16 (25.0%)	0.45
Diabetes	22 (19.1%)	13 (25.5%)	9 (14.1%)	0.12
Cardiovascular disease	56 (48.7%)	30 (58.8%)	26 (40.6%)	0.05
Osteoporosis	27 (23.5%)	9 (17.6%)	18 (28.1%)	0.19
Arthritis	23 (20.0%)	10 (19.6%)	13 (20.3%)	0.93
Asthma	22 (19.1%)	14 (27.5%)	8 (12.5%)	0.04
Sleep apnea	16 (13.9%)	11 (21.6%)	5 (7.8%)	0.03
Referred to pulmonary rehabilitation	74 (65.5%)	28 (56.0%)	46 (73.0%)	0.06

Abbreviations: CAT (COPD Assessment Test), FEV1 (forced expiratory volume in 1 s), FVC (forced vital capacity), LTOT (long term oxygen therapy), MRC (Medical Research Council dyspnea score), NIV (non-invasive ventilation), SD (standard deviation).

**Table 2:** Predictors of quality-of-life item in PRO questionnaire among patients with COPD (n = 115).

Predictor	Unstandardized Coefficients		Standardized Coefficients	*t*	*p*
	B	Std. Error	Beta		
Age	0.007	0.015	0.041	0.45	0.655
Gender					
Male	-	-	-	-	-
Female	−0.176	0.260	−0.060	−0.67	0.501
Smoking status					
Current	-	-	-	-	-
Former	−0.452	0.282	−0.147	−1.60	0.113
FEV1% pred.	−0.012	0.009	−0.122	−1.38	0.171
CAT	−0.097	0.020	−0.434	−4.91	0.000

Abbreviations: CAT (COPD Assessment Test), FEV1 (forced expiratory volume in 1 s).

## Data Availability

The raw data supporting the conclusions of this article will be made available by the authors upon reasonable request.

## Appendix A

**Table A1:** An overview of the items in the PRO-Pall tool. The translation from Danish to English has been performed by the research team, and the translation has not been psychometrically evaluated.

Item Number	Wording	Response Categories	Type of Symptom/Problem	Source
1	Do you have difficulty going for a short walk outdoors?	Not at all A little Somewhat A lot	Physical	EORTC-QLQ-C15 PAL
2	Do you have to lie in bed or sit in a chair during the day?	Not at all A little Somewhat A lot	Physical	EORTC-QLQ-C15 PAL
3	Do you need help eating dressing, washing or going to the toilet?	Not at all A little Somewhat A lot	Physical	EORTC-QLQ-C15 PAL
4	During the past week, were you short of breath?	Not at all A little Somewhat A lot	Physical	EORTC-QLQ-C15 PAL
5	During the past week, have you had pain?	Not at all A little Somewhat A lot	Physical	EORTC-QLQ-C15 PAL
6	During the past week, have you had trouble sleeping?	Not at all A little Somewhat A lot	Physical	EORTC-QLQ-C15 PAL
7	During the past week, have you felt weak?	Not at all A little Somewhat A lot	Physical	EORTC-QLQ-C15 PAL
8	During the past week, have you lacked appetite?	Not at all A little Somewhat A lot	Physical	EORTC-QLQ-C15 PAL
9	During the past week, have you felt nauseous?	Not at all A little Somewhat A lot	Physical	EORTC-QLQ-C15 PAL
10	During the past week, have you been constipated?	Not at all A little Somewhat A lot	Physical	EORTC-QLQ-C15 PAL
11	During the past week, were you tired?	Not at all A little Somewhat A lot	Physical	EORTC-QLQ-C15 PAL
12	During the past week, did pain interfere with your daily activities?	Not at all A little Somewhat A lot	Physical	EORTC-QLQ-C15 PAL
13	During the past week, did you feel tense?	Not at all A little Somewhat A lot	Psychosocial	EORTC-QLQ-C15 PAL
14	During the past week, did you feel depressed?	Not at all A little Somewhat A lot	Psychosocial	EORTC-QLQ-C15 PAL
15	How would you rate your overall quality of life during the last week?	7 point Likert scale ranging from ‘Very poor’ to ‘Excellent’	Quality of life	EORTC-QLQ-C15 PAL
16	During the past week, did you have sore or dry mouth?	Not at all A little Somewhat A lot	Physical	New item
17	During the past week, have you had swelling in any part of the body?	Not at all A little Somewhat A lot	Physical	EORTC item library
18	During the past week, have you missed intimacy? (e.g., closeness, tenderness, sex)	Not at all A little Somewhat A lot	Psychosocial	EORTC item library
19	During the past week, I felt lonely	Not at all A little Somewhat A lot	Psychosocial	EORTC item library
20	Have you been worried about whether your role towards family or friends has changed?	Not at all A little Somewhat Alot	Psychosocial	New item
21	Have you had thoughts about life or your situation that you need to talk about?	Not at all A little Somewhat Alot	Existential	New item
22	Have the problems you have had in connection with your illness (such as financial, practical or personal) been taken care of?	Problems have been taken care of; Problems have mostly been taken care of; Problems have partly been taken care of; Problems have by and large not been taken care of; Problems have not at all been taken care of	Psychosocial	New item
23	Have you been able to share your feelings with your family or friends as much as you would like?	All the time Most of the time Sometimes Rarely Not at all	Psychosocial	New item
24	Have you had any other significant symptoms or problems not mentioned in the questions above?	No Yes. Please list the most important (up to three) and indicate the extent to which you have had the symptoms or problems in the last week	Any	WISP
